# Involvement of the endocannabinoid system in the inhibition of Sindbis virus replication: a preliminary study

**DOI:** 10.1186/s42238-021-00068-y

**Published:** 2021-04-23

**Authors:** Juan L. Rodriguez, Joseph A. Lopez, J. Jordan Steel

**Affiliations:** 1 Biology Department, Colorado State University-Pueblo, 2200 Bonforte Blvd LS220, Pueblo, CO 81001 USA; 2 Department of Biology, US Air Force Academy, 2355 Faculty Dr. DFB, Colorado Springs, CO USA

**Keywords:** Alphavirus, Cannabinoids, CB1, Sindbis virus, Arachidonyl-2-chloroethanolamide (ACEA), AM251

## Abstract

**Background:**

Sindbis virus (Alphaviridae) is a plus-strand RNA virus that is dependent on the host cell for replication. Cannabinoid (CB) receptors are found on most human cells, including virally infected cells. Activation of cannabinoid receptors has been shown to alter normal cellular physiology. This study aimed to assess how agonist (ACEA) or antagonists/inverse agonist (AM251) of the cannabinoid receptors would alter the cellular environment and impact Sindbis virus replication.

**Methods:**

Human hepatoma (Huh7) cells were used as our model for viral replication. Cells were infected with Sindbis virus (SINV) and then treated with CB agonist (ACEA) (10 μM) or antagonist/inverse agonist (AM-251) (10 μM) and virus replication was monitored. A double subgenomic Sindbis virus containing a green fluorescent protein (GFP) reporter gene inserted into a 3′ subgenomic promoter was utilized for these assays to quickly measure viral replication. GFP fluorescent cells were analyzed using flow cytometry to measure the percentage of cells expressing the viral reporter and also quantify the levels of GFP fluorescence.

**Result:**

Treatment of SINV-infected Huh7 cells with CB1 receptor antagonist/inverse agonist (AM251, 10 μM) resulted in a significant decrease in viral replication, while infected cells treated with a CB1 receptor agonist (ACEA, 10 μM) resulted in a significant increase of viral infection. The data indicates that activation of CB1 receptor by cannabinoids significantly influences the ability of Sindbis virus to replicate in the host cell.

**Conclusion:**

Blocking CB1 receptor activity with 10 μM AM251 reduced viral replication, but activating the CB1 receptor with 10 μM ACEA resulted in an increase in viral infection. These results indicate cannabinoids may significantly impact a virus replicating in human liver cells. Future confirmation with other viruses and cell lines will be performed to better understand the impact of cannabinoids on viral infections.

## Background

Alphaviruses, such as Sindbis virus (SINV), are plus-strand RNA viruses with an enveloped virion of about 70 nm diameter. Alphavirus is a broad genus of viruses that include Sindbis virus, chikungunya virus, Eastern, Western, and Venezuelan equine encephalitis virus, Ross River virus, and Semliki Forest virus. Alphaviruses infect millions of humans and animals each year (Strauss and Strauss 1994; de Oliveira Mota et al. 2016). They are mainly transmitted through mosquito vectors, which are rapidly spreading across the globe. Chikungunya virus (CHIKV) has recently gone through a global outbreak resulting in over 2.9 million confirmed and suspected cases and almost 300 fatalities during summer 2016 (Wahid et al. 2017; Luis and De Figueiredo 2014). Alphavirus infections result in a variety of diseases and symptoms including rash, malaise, chronic arthralgia, or even deadly encephalitis (Atkins 2013; Zacks and Paessler 2010; Sun et al. 2014). Nearly 50% of individuals infected with chikungunya virus report having severe and disabling joint pain even 3 years after the initial infection (Simon et al. 2015; Schilte et al. 2013; Marimoutou et al. 2012). Despite the millions of people infected with CHIKV and chronic arthralgia, there are no specific treatments or drugs commercially available for individuals infected with Alphaviruses and it is necessary to evaluate plausible treatment options, one of which, that targets the endocannabinoid system.

The medicinal benefits of targeting the endocannabinoid system have been studied for many decades and have compelling evidence of its clinical efficacy (Park and Wu 2017; Rong et al. 2017; Makriyannis and Rapaka 1987). Cannabinoids have been used to treat a wide variety of diseases and health conditions including physical, emotional, and neurological diseases (Ryan and Sharts-Hopko 2017; Shah et al. 2017). Specifically, cannabinoids have been shown to reduce inflammation and can lower joint pain caused by arthritis or other injury (Mbvundula et al. 2004). These clinical and medical findings are becoming more well-known and accepted, but the molecular and mechanistic reasoning for many of these treatments still remains unclear. Although a dampened inflammatory response caused by cannabinoids may help the individual feel better, the question remains how the cannabinoids directly affects the underlying condition. We are seeking to understand the impact of cannabinoids directly on cells infected with an alphavirus.

Many cells in the human body have cannabinoid (CB) receptors (Pertwee 2008; Pacher et al. 2006). There are two different cannabinoid (CB) receptors that have been found on human cells: CB1 and CB2 (Tuccinardi et al. 2006; Ai and Chang 2012). Neurons, lungs, liver, and other digestive and reproductive tissues have CB1, where CB2 is primarily found on neurons and cells associated with the immune system, including the spleen, bones, and lymphocytes (Liu et al. 2017; Stempel et al. 2016). These CB receptors bind to endogenous, exogenous, or synthetic cannabinoids and trigger specific cellular responses (Tuccinardi et al. 2006). The binding and stimulation of the CB receptors seems to be the key for the clinical and physiological responses induced during cannabinoid use. CB receptor agonists, such as arachidonyl-2-chloroethanolamide (ACEA), bind to CB1 receptors and activate signaling pathways (Ai and Chang 2012; Biernacki and Skrzydlewska 2016; Ma et al. 2015). Antagonists/inverse agonists, such as AM-251, bind and block the stimulation of the receptor and subsequent cellular pathways (Shahidi et al. 2014; Chanda et al. 2011). These synthetic CB1 receptor ligands are powerful tools for studying cannabinoid-induced cellular changes.

Activation of CB1 receptors stimulates fatty acid synthesis and gluconeogenesis in human liver cells (Chanda et al. 2011; Osei-Hyiaman et al. 2005a). This elevated metabolic phenotype has been shown to play roles in obesity and in viral hepatitis (caused by Flaviviruses) (Pacher et al. 2006; Osei-Hyiaman et al. 2008; Toyoda et al. 2011; Shahidi et al. 2014; Osei-Hyiaman et al. 2005b; Agilent 2015). Viruses are completely dependent on the infected host cell for energy and metabolites to synthesize and produce new virions. Many viruses alter the host cell physiology and elevate certain metabolic pathways to enhance the virus replication rate (Sanchez and Lagunoff 2015; Fontaine et al. 2014; Heaton and Randall 2011). Alphaviruses, including Sindbis virus, have been shown to require glycolysis for an optimal replication cycle and without sufficient glucose metabolism, the virus is inhibited (Findlay and Ulaeto 2015; Silva da Costa et al. 2012). These data indicate that both CB1 receptor stimulation and virus infection result in altered cellular physiology. CB1 receptor activation increases metabolism, specifically through a gluconeogenesis pathway which could enhance Sindbis virus infection which relies on glycolytic activity (Chanda et al. 2011). Gluconeogenesis and glycolysis are both glucose metabolic pathways although essentially opposite from each other with gluconeogenesis being an anabolic pathway and glycolysis being catabolic, there could be benefits to viral replication for the synthesis of glucose. We hypothesized that CB1 receptor deactivation and Sindbis virus infection would suppress glycolytic cellular pathways that would result in non-optimal conditions for virus replication and would successfully inhibit viral infection. Previous reports have shown that cannabinoids can affect viral infection, including Kaposi sarcoma-associated herpesvirus, herpes simplex virus, human immunodeficiency virus, and hepatitis (Medveczky et al. 2004; Reiss 2010; Maor et al. 2012). In this study, we have verified that SINV infects liver cells and have investigated the impact of cannabinoid receptor stimulation on Sindbis virus (alphavirus) replication.

## Methods

### Cells and cell culture

Human hepatoma (Huh7) cells were cultured using DMEM media containing streptomycin/penicillin and FBS (10%). Huh7 cells are adherent and were cultured in T75 flasks or 24-well culture plates in an incubator at 37 °C and 5% CO_2_. Cells were monitored daily and at ~75% confluence, the cells were passaged by washing with PBS and treating with 0.25% trypsin. Cells were counted with a hemocytometer and seeded into well plates 24 h before an experiment so that the cells would be nearly 70% confluent at the time of infection/treatment. Baby hamster kidney (BHK) cells and human embryonic kidney (HEK) cells were maintained in similar conditions. Expression of CB1R was verified in cultured cells by immunofluorescence assay with a primary antibody (1AB) rabbit anti-cannabinoid receptor 1 antibody (Abcam. Ab237303) and a secondary, goat polyclonal secondary antibody to rabbit IgG Alexa Fluor 488 (Abcam. Ab150077)

### Virus and viral infection

A double sub-genomic promoter Sindbis virus (dsSINV) expressing a green fluorescent protein (GFP) at the 3′ end of the viral genome was used in the experiments. Sindbis virus (SINV) belongs to the *Togaviridae* family and *alphavirus* genus of viruses. SINV is a typical prototype alphavirus, which was used as the model virus for all experiments. The GFP reporter is inserted into the viral genome and GFP fluorescence serves as an indicator for viral replication in infected cells. Double sub-genomic alphavirus reporters have been used for several years in alphavirus research and are great reporter viruses used in gathering data on virus replication. To infect cultured cells, the cells were removed from the plate using trypsin, suspended, and counted with trypan blue staining and a hemocytometer. The resulting cell concentration (cells/well) was used to calculate how much virus to add to each well. Cells were infected with a multiplicity of infection (MOI), which is the ratio of infectious virus to cell, of 1 or otherwise stated. Virus was taken from a stock vial and added to culture media and then placed on the cells and incubated for 1 h at 37 °C, which is sufficient time for the virus to enter the host cells. After 1 h of infection, the virus media was replaced with fresh media. This fresh media could contain the agonist/antagonist treatment. The infected cells were incubated for the specified time, usually 24 h, to allow the virus to infect the cells.

### Agonist/antagonist treatment

Arachidonyl-2-chloroethanolamide (ACEA) and AM-251 were purchased from Tocris Bioscience. The compounds were suspended in DMSO at a 100 mM stock concentration. The 100 mM stock was diluted with solvent to generate stock concentrations at 50 mM, 25 mM, 12.5 mM, 6.25 mM, 3.125 mM, and 1.56 mM. To treat the cells, the mM stocks were diluted at 1:1000, resulting in μM concentrations in cell culture media that could be added directly to the infected or mock-infected cells. A mock/control treatment was prepared by diluting only solvent (DMSO) 1:1000 in cell culture media. The dilutions of ACEA or AM-251 in cell culture media were always prepared fresh prior to setting up an experiment.

### Cytotoxicity of ACEA/AM-251 and plate reader analysis

Cytotoxicity and cell viability were measured with the Alamar blue Cell Viability Assay® from ThermoFisher according to manufacturer’s protocols. Briefly, cells were stained with a 100-μM concentration of Resazurin/Alamar blue and incubated for 1 h at 37 °C. After 1 h, the resazurin would be broken down to the fluorescent resorufin in living cells and the plates were analyzed on an EnSpire Multimode Plate Reader® from PerkinElmer for fluorescence at an excitation of 540-570 nm and an emission of 580-610 nm to measure the living cells. The mock-/control-treated cells were set to 100% and then the viability measurements from treated wells were calculated as a percentage of the control. All experiments were performed and analyzed multiple times in triplicate.

### Quantifying virus replication and flow cytometry

Virus replication/infection was measured by looking at GFP fluorescence from the reporter inserted into the viral genome. Cells were analyzed using a Guava easyCyte® flow cytometer from Millipore. Briefly, cells were trypsinized, resuspended, and run over the flow cytometer to quantify the number and percent of cells infected (expressing GFP) and the relative levels of GFP fluorescence per cell. In total, 10,000 cells were analyzed for each sample and each condition was run in triplicate on the flow cytometer. The same settings (gains, parameters, regions, thresholds, and gating) were used for all analysis to accurately quantify and measure dsSINV-GFP positive cells. Data from the flow cytometer allowed specific analysis of GFP fluorescence per cell, number of cells infected, percent infected, etc.

### Statistical analysis section

Statistical significance was determined using a one-way ANOVA followed by a Tukey’s post hoc analysis.

## Results

### Confirmation that Huh7 model cell line

**Fig. 1:**
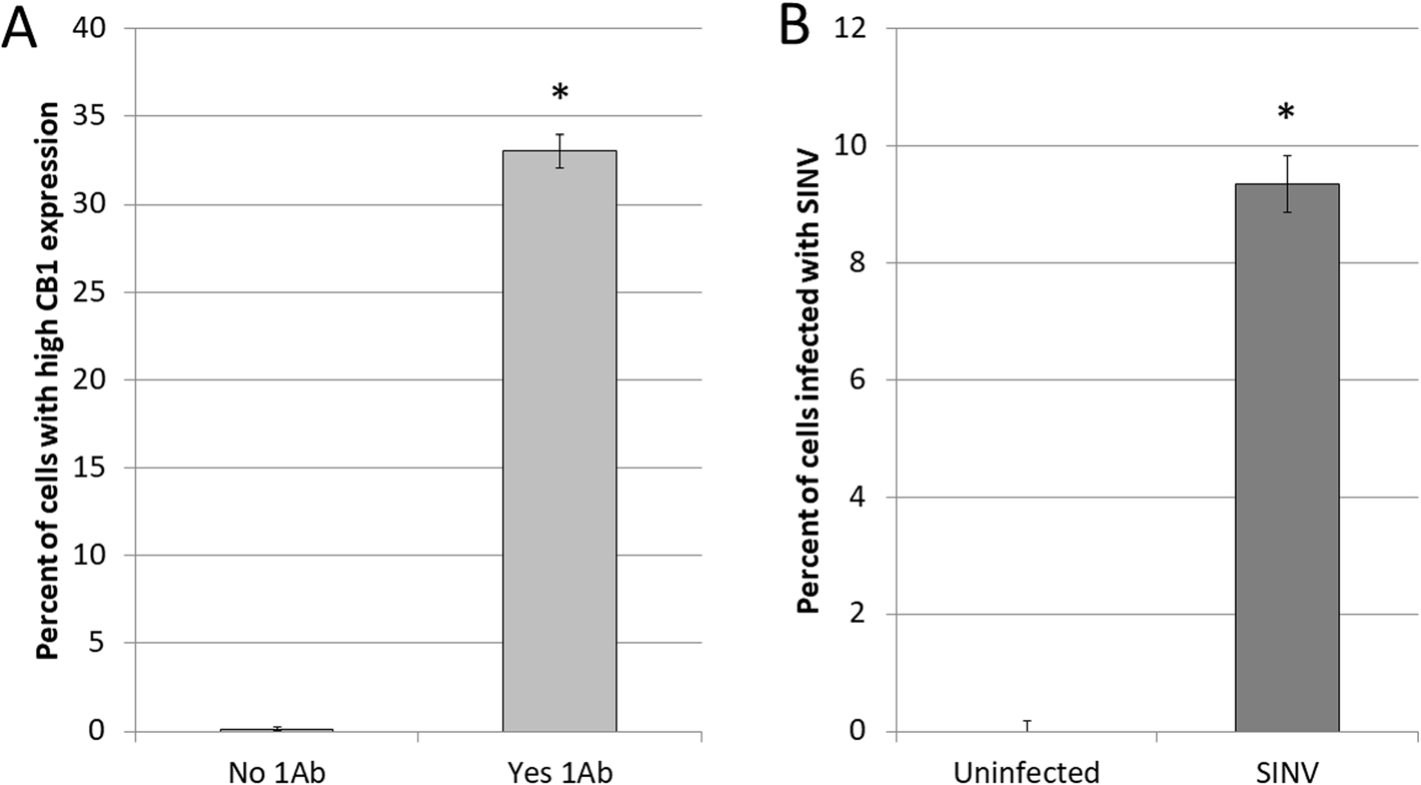
**a** Validation CB1 receptor expression in human liver (Huh7) cell line. Cells were exposed to a primary antibody (1Ab) that binds specifically to the cannabinoid receptor I (ab23703). The antibody is conjugated with a fluorophore for detection and quantification. Cells with no 1Ab, did not give off any signal, whereas cells exposed to the primary antibody-targeting CB1 resulted in high level of detection, indicating a strong presence of the CB1 receptor. **b** Human liver (Huh7) cells can successfully be infected with Sindbis virus. After 24 h, nearly 10% of cells are infected with Sindbis virus

To investigate our hypothesis and determine the impact of cannabinoids on alphavirus replication, we utilized a human liver cell line (Huh7). Confirmation of CB1 receptor expression on Huh7 cells was verified through an immunofluorescent assay (Fig. 1a). Viral infection requires that a host cell is both susceptible for entry and permissive for viral replication. We confirmed that Huh7 cells are susceptible and permissive to Sindbis virus infection by infecting them with a double subgenomic Sindbis virus that expresses a green fluorescent protein (GFP). At 24 h post infection, 10% of cells were infected and rapidly expressing the GFP viral marker (Fig. 1b). Therefore, Huh7 cells are susceptible and permissive to Sindbis virus replication and also express CB1 receptor and can be used to assess how CB1 stimulation affects SINV replication

### CB1 receptor antagonist and agonist cytotoxicity

**Fig. 2:**
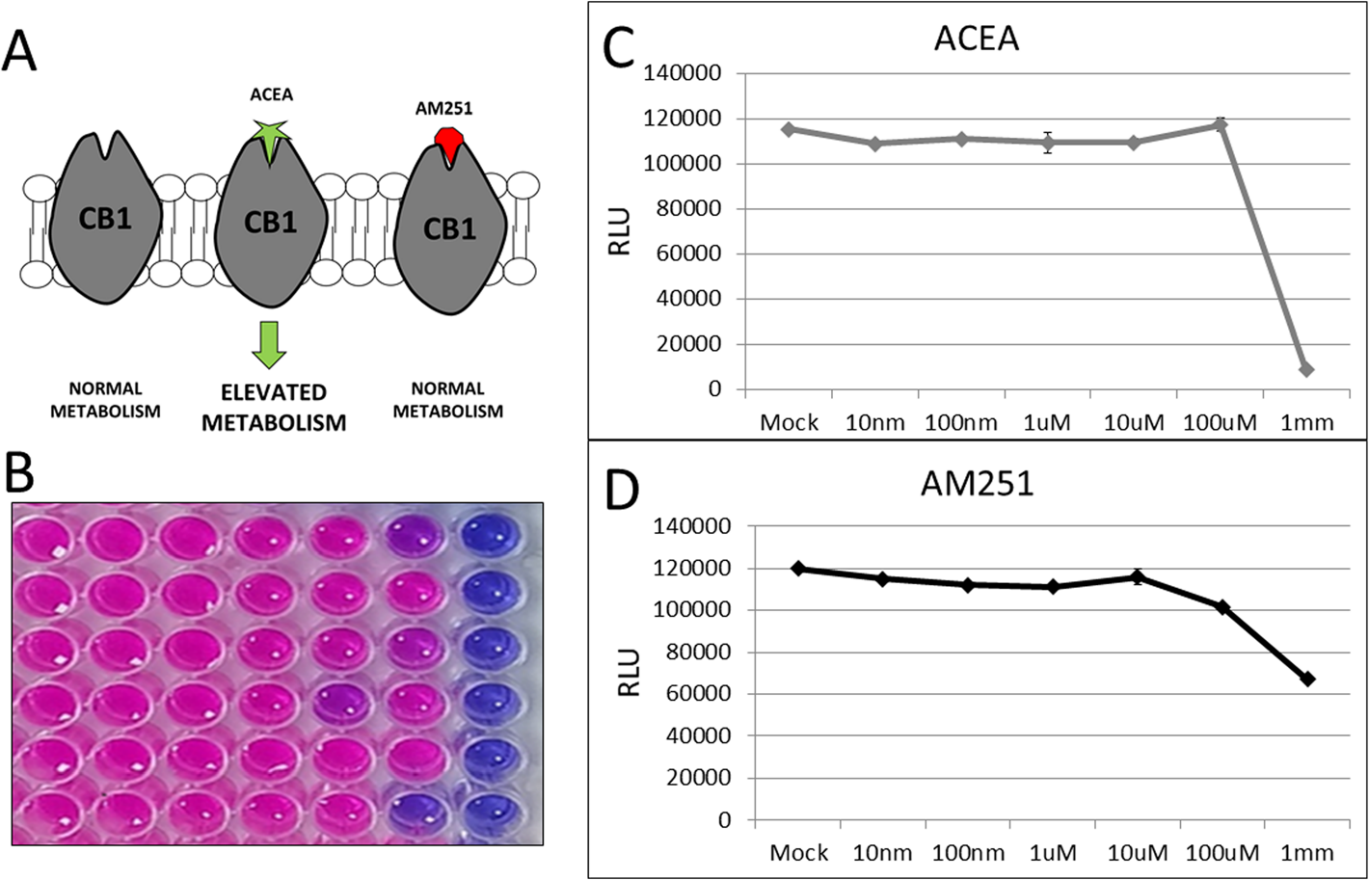
**a** CB1 agonist/antagonist diagram illustrating the synthetic cannabinoid compounds used in this study. **b** Resazurin viability stain to indicate cytotoxicity effects of the cannabinoid treatment. **C** and **d** Quantification of cytotoxicity data shown in (**b**). ACEA and AM251 treatment is not toxic to the cells until high concentrations of 1 mM. Treatment with 10 μM have no adverse cytotoxicity effects

Arachidonyl-2-chloroethanolamide (ACEA) has been shown to be an effective agonist for CB1 receptors. The cannabinoid binds to the CB1 and transducing a signaling pathway through a family of G proteins and into the cytoplasm of the effected cell. AM-251 is an antagonist/inverse agonist to CB1 receptors and successfully blocks the receptor from being activated. Adding a ligand or compound to cell media can have off-target or cytotoxic effects to cultured cells. Cell viability was monitored by treating cells with increasing concentrations of either ACEA or AM251 and measuring their viability using the Alamar blue assay. For ACEA treated cells, there was no cytotoxicity observed until concentrations reached into the mM range. For AM-251, cells were healthy until concentrations reached about 100 μM, and then concentrations above 100 μM started to inhibit cell viability (Fig. 2). Ten micromolars was determined to be a high concentration of the ligand without inducing cytotoxicity and is used for all subsequent experiments.

### ACEA treatment of SINV-infected cells

**Fig. 3:**
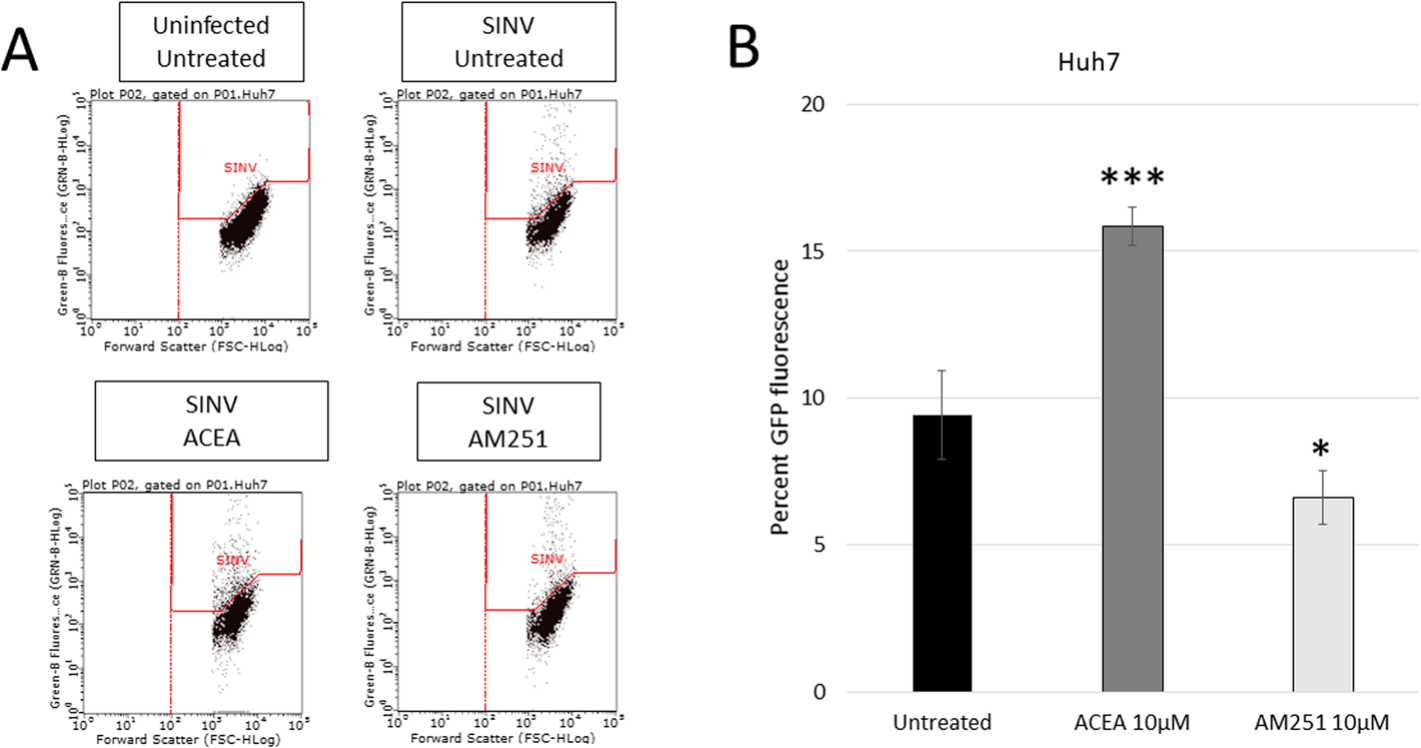
Effect of ACEA/AM251 treatment on SINV-infected Huh7 cells. (**a**) Flow cytometry data of uninfected and Sindbis virus infected cells that are untreated, ACEA treated (10 μM), or AM251 treated (10 μM). Each dot represents a single cell in the assay (**b**) quantification of results from 3A. ACEA treatment increased the cells that are infected and AM251 decreased the cells that are infected

Huh7 cells were infected with dsSINV-GFP at an MOI of 1, and after 1 h of infecting the cells were treated with ACEA, AM-251, or solvent only. After 24 h of infection, the cells were collected and analyzed for the level of GFP fluorescence, indicating the level of viral infection. Flow cytometry analysis confirms the viral infection due to the shift of GFP fluorescent cells into the designated “SINV” region for infected cells. All samples had similar percentages of infected cells due to the same infection MOI, time, and conditions. However, after 24 h of infection, analysis of the level of GFP expression, not the percentage of green fluorescent cells, but the level or intensity of infection, indicates some significant differences. AM251 treatment (10 μM) reduced viral replication significantly compared to the untreated control, while ACEA treatment (10 μM) increased viral replication (Fig. 3). This lower level of GFP fluorescence indicates slower or perhaps inhibited viral replication in cells with a deactivated CB1 receptor.

### Validation in other cell types

**Fig. 4:**
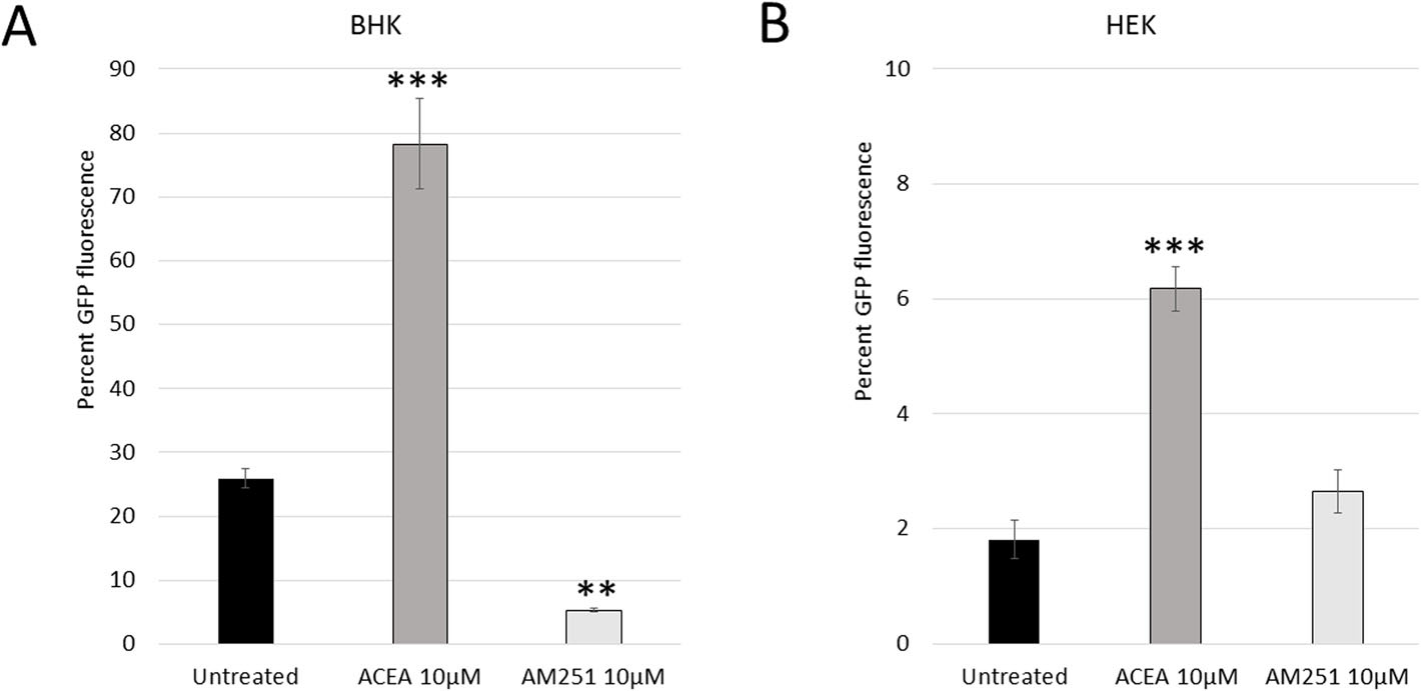
Confirmation of ACEA/AM251 treatment on SINV-infected baby hamster kidney (BHK) and human embryonic kidney (HEK) cells. Results from flow cytometry analysis of individual cells. Confirms results in Fig. 3 with two additional cell types

The AM251-induced reduction in SINV replication was further confirmed in other cell lines. Human embryonic kidney (HEK) cells and baby hamster kidney (BHK) cells are traditional model cell lines used in virology research and are susceptible and permissive to a wide variety of viruses. Both cell lines express the CB1 receptor and have similar cytotoxicity thresholds with ACEA and AM251 (data not shown). Although the cell lines infected at different efficiencies, both cell lines displayed a similar trend in viral replication when being treated with ACEA or AM-251 (Fig. 4). ACEA significantly increased viral infection, with more than two times the amount of infection in ACEA-treated cells, and AM251 treatment consistently reduced viral replication when compared to untreated SINV-infected cells.

## Discussion

Viruses are the most abundant pathogen on the earth and are responsible for significant human infection and disease. As obligate intracellular parasites, viruses are completely dependent on the host cell for survival. Drugs or stimuli that affect cells will directly affect viruses that are attempting to infect and use the cell as a host. As medicinal cannabinoids become more and more utilized, it is important to understand how activation of cannabinoid receptors affects viral infections. THC and CBD are commonly used medicinally and recreationally and it is critical to know the impact of cannabinoid receptor activation and any implications to increasing or decreasing the risk of viral infections. While this study did not use THC and CBD directly, the compound ACEA serves as an agonist and activator for cannabinoid receptors and serves as a model for what may occur with other cannabinoids.

Our results confirm that cultured human liver cells (Huh7) express CB1 and are capable of being infected by Sindbis virus (Fig. 1). Treating cells with CB1 receptor agonist (ACEA) or antagonist (AM251) had minimal cytotoxicity effects until high concentrations were reached around the 1 mM level (Fig. 2). Cells have these receptors to respond to normal endocannabinoids within the body, so it is not surprising that these compounds are non-toxic at low concentrations. The cytotoxicity that is observed at high concentrations may not be to over stimulation of the normal CB1 activation pathway, but rather, due to the abundance of the ligand and off-target binding to other receptors and subsequent signaling.

CB1 activation in liver cells has been previously reported to induce fatty acid synthesis and gluconeogenesis, which are important cellular anabolic pathways involved in building glucose and synthesizing biomolecules. Alphaviruses, in a similar fashion, have been described as utilizing bioenergetic pathways, to support macromolecule synthesis. Although both CB1 stimulation and virus infection have been reported to alter and increase normal cell metabolism, this is the first-time reporting what happens when both cell stimulants are occurring at the same time with alphaviruses. Huh7 cells that were infected with SINV and then treated with AM251 displayed a significant reduction in virus replication when compared to mock-treated or ACEA-treated cells (Fig. 3). This is most likely because after the virus enters the cell, it begins to hijack and manipulate the cell for its optimal conditions, but the stimulation of the CB1 receptor deactivates alternative cellular pathways that reduced the available resources within the cell. With limited resources, the virus replication is inhibited (Fig. 3). This reduction in viral infection represents a significant finding and indicates that cannabinoids may be effective in reducing viral burdens in those that are infected.

These results were validated in two other cell lines, HEK and BHK cells, where AM251 treatment reduced viral replication similar to the Huh7 data. These findings are very promising, but further analysis and confirmation are definitely needed to verify and explore cannabinoid’s anti-viral effects. Viruses are very different from each other, especially in different genus’s or families, but even at the species to species level, so careful analysis will need to be performed to investigate how cannabinoids impact virus replication for a wide variety of viruses. Nonetheless, these initial results from this pilot study are very intriguing; AM251 treatment significantly reduced viral replication for Sindbis virus in human liver cells, while ACEA treatment increased viral infection. The strengths of this preliminary study are that despite only using 2 synthetic cannabinoids in a cell model system, the results are very robust and have been validated through repeat experiments and in different types of cells. Although more work needs to be done with actual living organisms and with other viruses and cannabinoids, this pilot study presents some rigorous data that highlights a very intriguing and critical need to investigate how cannabinoids affect infectious pathogens and other cellular functions.

## Conclusions

Stimulation of the CB1 receptor by treatment with 10 μM ACEA, resulted in an increase in Sindbis virus replication. Treatment with 10 μM AM251, a reverse agonist of CB1 receptor reduced viral infection.

## Data Availability

The datasets used and/or analyzed during the current study are available from the corresponding author on reasonable request.

## Abbreviations

CB1R: Cannabinoid receptor 1
CB2R: Cannabinoid receptor 2
SINV: Sindbis virus
CHIKV: Chikungunya virus
dsSINV-GFP: Double subgenomic SINV expressing GFP
GFP: Green fluorescent protein
ACEA: Arachidonyl-2-chloroethanolamide
MOI: Multiplicity of infection
hpi: Hours post infection
